# Anthracycline-induced cardiotoxicity and renin-angiotensin-aldosterone system—from molecular mechanisms to therapeutic applications

**DOI:** 10.1007/s10741-020-09977-1

**Published:** 2020-05-30

**Authors:** Paweł Sobczuk, Magdalena Czerwińska, Marcin Kleibert, Agnieszka Cudnoch-Jędrzejewska

**Affiliations:** 1 Department of Experimental and Clinical Physiology, Laboratory of Centre for Preclinical Research, Medical University of Warsaw, Warsaw, Poland; 2 Department of Soft Tissue/Bone Sarcoma and Melanoma, Maria Sklodowska-Curie National Research Institute of Oncology, Warsaw, Poland

**Keywords:** Doxorubicin, Cardiotoxicity, Angiotensin, RAAS, ACEI, ARB

## Abstract

Few millions of new cancer cases are diagnosed worldwide every year. Due to significant progress in understanding cancer biology and developing new therapies, the mortality rates are decreasing with many of patients that can be completely cured. However, vast majority of them require chemotherapy which comes with high medical costs in terms of adverse events, of which cardiotoxicity is one of the most serious and challenging. Anthracyclines (doxorubicin, epirubicin) are a class of cytotoxic agents used in treatment of breast cancer, sarcomas, or hematological malignancies that are associated with high risk of cardiotoxicity that is observed in even up to 30% of patients and can be diagnosed years after the therapy. The mechanism, in which anthracyclines cause cardiotoxicity are not well known, but it is proposed that dysregulation of renin-angiotensin-aldosterone system (RAAS), one of main humoral regulators of cardiovascular system, may play a significant role. There is increasing evidence that drugs targeting this system can be effective in the prevention and treatment of anthracycline-induced cardiotoxicity what has recently found reflection in the recommendation of some scientific societies. In this review, we comprehensively describe possible mechanisms how anthracyclines affect RAAS and lead to cardiotoxicity. Moreover, we critically review available preclinical and clinical data on use of RAAS inhibitors in the primary and secondary prevention and treatment of cardiac adverse events associated with anthracycline-based chemotherapy.

## Introduction

Recent years have brought extensive and fast changes in the landscape of oncological treatment with new targeted drugs and immunotherapy approved for clinical use. However, cytotoxic chemotherapy remains a standard of care in various cancer types. One of the main concerns associated with chemotherapy are the adverse and toxic effects. While we have learned how to manage early onset toxicities, like nausea or vomiting, long-term treatment-related toxicities are still not fully understood. Cardiotoxicity, often caused by anthracyclines, is probably one of the most important and threatening events that require more interest.

Doxorubicin (DOX) belongs to anthracycline family, along with epirubicin, daunorubicin, and idarubicin [1]. Anthracyclines are well-established and highly effective anti-neoplastic agents, used to treat several adult and pediatric cancers, such as breast cancer, leukemia, lymphomas, sarcomas, and many others. Retrospective analyses of patients treated with anthracyclines showed that many patients experienced cardiotoxicity.

Anthracycline-induced cardiotoxicity (AIC) can occur as acute or chronic, and is characterized by a broad spectrum of symptoms including asymptomatic electrocardiography (ECG) changes as arrhythmia, cardiomyopathy, pericarditis, left ventricular dysfunction, and decompensated heart failure [2]. There is no consensus on definitions of AIC and different research groups and societies adapt their own ones. Commonly, cardiotoxicity is defined as presence of signs and symptoms of heart failure or asymptomatic decline of left ventricular ejection fraction (LVEF) ≥ 10% to final LVEF < 55%. However, some clinical trials have adapted modified criteria, thus special caution is necessary when comparing the results [3, 4].

Acute cardiotoxicity is rare, not dose-related and often associated with preexisting cardiac diseases [5]. More common and by far more serious is chronic cardiotoxicity, which usually manifests during the first year after the end of anthracycline treatment but can also occur decades later. The risk of developing cardiotoxicity increases with the cumulative dose of doxorubicin—with 400 mg/m^2^, there is a 5% incidence of cardiotoxicity, with 550 mg/m^2^ 26% risk, and with 700 mg/m^2^, the risk is as high as 48% [6]. Breast cancer patients treated with doxorubicin showed decreased LVEF when the cumulative doxorubicin dose exceeded 350 mg/m^2^ [7]. In a retrospective study comprising 4000 patients, the incidence ranged from 0.1 to 18.0%, depending on the cumulative dose [8]. In consequence, reduction of the maximum cumulative dose to 550 mg/m^2^ was recommended, which unfortunately is accompanied by reduced anti-tumor efficacy. However, despite the success to drop early-onset events, no reduction of late-onset complications has been observed, launching the notion that there is no dose of DOX to be considered completely safe [9, 10].

Important observations came from the follow-up of childhood cancer survivors who were treated with DOX. Echocardiographic abnormalities were detected in up to 50% of them [11]. About 10% of these patients will develop symptomatic cardiomyopathy up to 15 years after the end of chemotherapy [12]. Moreover, childhood cancer survivors, treated with anthracyclines and radiotherapy, have a high-risk of cardiac events at an early age, and a 12.5% risk of developing heart failure (HF) within 30 years after treatment [13]. Of note, the use of myocardial strain imaging, as an alternative to standard echocardiography, demonstrated even higher prevalence of cardiac dysfunction associated with anthracycline treatment reaching up to 30% of adult survivors of childhood cancer [14].

Considering this, many researchers were trying to solve the problem of AIC pathogenesis and find effective strategies for prevention. Dysregulation of the renin-angiotensin-aldosterone system (RAAS) has been proposed to play a role in development of anthracycline-induced cardiac toxicity. Various preclinical and clinical studies showed that use of different RAAS inhibitors prevents from AIC. In this review, we summarize current knowledge about how anthracyclines affect RAAS and present data from preclinical and clinical research on targeting RAAS for prevention and treatment of cardiotoxicity. Combining data from preclinical research, mostly from animal models, and clinical prospective and retrospective studies we have tried to analyze whether RAAS inhibitors can be use in the treatment or prevention of AIC, what is the optimal strategy for their use—primary or secondary prevention—and finally underline gaps and perspectives in this field.

## Mechanism of anthracycline-induced cardiotoxicity

Doxorubicin acquaintances robustly with cellular nuclei and intercalate with DNA bases to mediate doxorubicin-DNA complexes, ensuing in cell demise [15]. Generally, anthracyclines intercalate into DNA, form bulky DNA adducts and DNA crosslinks, which interfere with DNA replication and transcription [16]. Doxorubicin’s, like other anthracyclines’, main cellular target is a topoisomerase-II (Top2). Doxorubicin binds both DNA and Top2 to form the ternary Top2-doxorubicin-DNA cleavage complex, thereby causing DNA double-strand breaks (DSBs), which trigger the cell death if left unrepaired. There are two forms of Top2 enzymes: Top2α and Top2β. Top2α, a known marker of cell proliferation, is overexpressed in the tumor cells but is not detectable in quiescent tissues, contrary to Top2β. Action on Top2α is thus thought to be the molecular basis of doxorubicin’s anticancer activity [17, 18].

Besides interactions with topoisomerase, anthracyclines can damage DNA directly due to the generation of reactive oxygen species (ROS), leading to nucleotides oxidation, base mismatches, point mutations, and DNA single-strand breaks. The production of ROS also causes a DNA damage-independent stimulation of cytotoxic mechanisms, resulting from oxidative protein modifications, in particular, lipid peroxidation. Last, anthracyclines interfere with DNA helicase activity and DNA strands separation [19, 20].

Unfortunately, the geno- and cytotoxic effects evoked by anthracyclines are not limited to tumor cells. The successful use of doxorubicin has been hampered by toxicities such as hematopoietic suppression, nausea, vomiting, extravasation, and alopecia. Most of those effects are temporary and resolve after cessation of the therapy. However, significant proportion of patients may develop cardiac toxicities that may cause long-lasting effects, decrease survival, and affect quality of life.

The precise mechanism of doxorubicin-induced cardiotoxicity is still elusive. Myocardial damage induced by doxorubicin is considered as the key mechanism with the influence of ROS and Top2β as a key factor causing it. Primarily, it was believed that cardiotoxicity is mostly associated with generation of ROS while metabolizing anthracyclines. Anthracyclines undergo univalent reduction via enzymatic pathway involving NADH dehydrogenase (complex I) of the mitochondrial electron transport chain to a semiquinone radical which in presence of molecular oxygen is further proceeded into a superoxide anion [21, 22]. ROS may also be produced from doxorubicin-iron complexes that can form toxic radical and reactive nitrogen species, resulting in increased nitrosative stress and mitochondrial dysfunction [23]. It is generally accepted that the oxidative stress leads to the activation of molecular pathways causing the loss of cardiomyocytes through necrosis and apoptosis [24, 25]. Convincing results about the entity and occurrence of apoptosis raised the possibility that apoptotic-related mechanisms are central in the setting of acute cardiotoxicity but marginal in a scenario of chronic cardiomyopathy and HF [24]. Increasing evidence indicates that other mechanisms, including senescence or autophagy, take part in the anthracycline-driven cardiotoxic effects, affecting the functional activity of cardiomyocytes [26, 27] and stem cell populations [28]. The increased ROS generation and reduction of antioxidant capacity of cardiomyocytes is undoubted, however the hypothesis about the central role of ROS in AIC pathogenesis has been tempered by a series of studies, in which treatment with a ROS scavenger failed to prevent cardiac toxicity caused by doxorubicin [12, 29]. Thus, other mechanism has been discovered, what changed the perspective on molecular background of AIC, which is more complexed than though before.

Nowadays, interactions of anthracyclines with topoisomerases seem to have major impact on the molecular mechanisms of AIC. Adult mammalian cardiomyocytes do not express Top2α, the main target of anthracyclines, but express Top2β isoform. The knockout of Top2β gene in mice resulted in a significant reduction of doxorubicin-induced cardiomyocyte death suggesting that Top2β also serve as a target for doxorubicin. The Top2β-doxorubicin-DNA ternary cleavage complex can induce DNA double-strand breaks (DSBs), leading to cardiomyocytes death Moreover, Top2β works as a regulator of various genes expression, including the ones involved in mitochondrial biogenesis and antioxidant function [30]. Based on those observations, interactions between anthracyclines and Top2β are currently considered one of the key elements in the pathogenesis of cardiotoxicity.

Additional mechanisms contributing to doxorubicin cardiotoxicity are dysregulation of iron regulatory proteins, nitric oxide (NO) release, mitochondrial dysfunction, impaired adenosine triphosphate (ATP) level, hampered cardiac progenitor cells, dysregulation of calcium metabolism, release of inflammatory mediators, activation of ubiquitin protease system, and endothelial dysfunction [6].

Considering the variety of mechanism involved in AIC and promising observations from preclinical and clinical studies with RAAS inhibitors it seems that dysregulation of RAAS may also occur during AIC development and significantly contribute to myocardial damage.

## Renin-angiotensin-aldosterone system

**Fig. 1:**
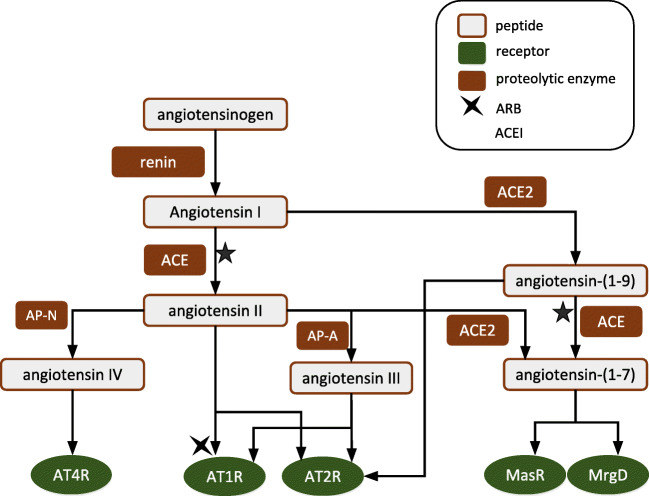
Schematic presentation of renin-angiotensin system. Light gray rectangle boxes represent appropriate polypeptides; dark rectangle boxes, proteolytic enzymes; dark oval boxes, receptors. Angiotensinogen is cleaved by renin to angiotensin I, which is further converted by angiotensin-converting enzyme (ACE) to angiotensin II, or by angiotensin converting enzyme type 2 (ACE2) to angiotensin-(1–9). Angiotensin II can act on its receptors: type 1 (AT1R) or type 2 (AT2R). It can be also further processed by neuropeptidase A (AP-A) to angiotensin III that acts on the AT1R and AT2R, by neuropeptidase N (AP-N) to angiotensin IV that has its own angiotensin receptor type 4 (AT4R), or by ACE2 to angiotensin-(1–7). Angiotensin-(1–7) exerts its function via two receptors: MasR and MrgD. ACE activity can be blocked by angiotensin-converting enzyme inhibitors (ACEI)—marked as a X sign, while angiotensin receptor blockers (ARB) inhibit function of AT1R—marked as a star

Renin-angiotensin-aldosterone system is a complex of polypeptide axes (Fig. 1) that play a central role in several physiological process such as cardiovascular regulation or hydro-electrolyte balance, but at the same time are involved in pathophysiology of many diseases like arterial hypertension, heart failure, and even cancer [31]. The basic component of RAAS is angiotensinogen (alpha-2-globulin, ATG) that is mainly produced and secreted by the liver. Further, renin—a proteinase mainly released by the juxtaglomerular apparatus of the kidney—degrades ATG to angiotensin I (ang-I). Release of renin can be triggered by the decrease in the plasma sodium ions concentration, decreased blood volume or low blood pressure. Ang-I is physiologically inactive and need to be converted to angiotensin II (ang-II) by angiotensin-converting enzyme (ACE). Highest quantities of ACE are found in the endothelial, epithelial, and neuroepithelial cells. Ang-II is the most active RAAS component and interacts with two types of G protein coupled receptors: angiotensin type 1 receptor (AT-1R) and angiotensin type 2 receptor (AT-2R) [32, 33]. Alternative RAAS pathway give a possibility to alternatively originate ang-II from ang-I through the activity of chymase, cathepsin G or CAGE, without involvement of ACE [34, 35]. Most of ang-II effects, including vasoconstriction, elevation of aldosterone plasma level, sustaining sodium and water, maintaining fluid and salt homeostasis, or increase of blood pressure are mediated by AT-1 receptor (Table 1) [36, 37]. Moreover, ang-II via AT-1R can induce processes such as fibrosis, inflammation, cardiac hypertrophy and reactive oxygen species production [38–41]. The AT-2R receptor, comparing to AT-1R, has mostly contrary function especially causing vasodilatation, natriuresis and inhibition of cell proliferation, however it is present mostly in the prenatal period and in adults its role is highly limited [42].

**Table 1:** Effects mediated by different angiotensin receptors

Angiotensin II via AT-1R	Angiotensin II via AT-2R	Angiotensin-(1–7) via MasR	Angiotensin IV via AT-4R
Vasoconstriction	Vasodilatation	Vasodilatation	Vasodilatation
Increased blood pressure	Decreased blood pressure	Decreased blood pressure	Decreased blood pressure
Water and sodium retention	Increased natriuresis and water removal	Increased natriuresis and water removal	Increased natriuresis and water removal
Increased renal cortical flow	Decreased renal cortical blood flow	Decreased renal cortical blood flow	Increased renal cortical blood flow
Proinflammatory effects	Anti-inflammatory effects	Anti-inflammatory effects	Anti-inflammatory effects
Decreased NO synthesis	Increased NO synthesis	Increased NO synthesis	Increased NO synthesis
Cardiac hypertrophy	Inhibition of cardiac hypertrophy	Inhibition of cardiac hypertrophy	Inhibition of cardiac hypertrophy
Induce cardiac fibrosis	Inhibition of cardiac fibrosis	Inhibition of cardiac fibrosis	
Decreased baroreceptors sensitivity	Increased baroreceptors sensitivity	Increased baroreceptors sensitivity	
Proangiogenic effect	Antiangiogenic effect	Antiangiogenic effect	
Proliferative effect	Antiproliferative effect	Antiproliferative effect	
Aldosterone and vasopressin release	Vasopressin release		
Decreased parasympathetic tone		Increased parasympathetic tone	
Increased sympathetic tone		Decreased sympathetic tone	
Increase of reactive oxygen species production			
Increased heart contractility			
Inhibition of renin release			
Increased expression of adhesion molecules			

ACE/ang-II/AT-1R axis is considered as classical in the RAAS. Nevertheless, ang-II can be also modified by aminopeptidases A or N to angiotensin III (ang-III) or angiotensin IV (ang-IV), respectively. Ang-III acts through the same receptors as ang-II, contrary to ang-IV which signals through their own receptor AT4-R leading to increase of renal cortical and cerebral blood flow and natriuresis (Table 1) [43–45]. There are also some reports that ang-IV inhibits apoptosis and inflammation in myocardial reperfusion injury [46].

Besides conventional ACE/ang-II/AT-1R axis, more attention is paid recently to alternative ACE2/ang-(1–7)/MasR axis. Carboxypeptidase ACE2, known as homolog of traditional ACE, is expressed mainly in the kidney, heart and testis. ACE2 main function is a conversion of ang-I to inactive angiotensin-(1–9) which further can be processed to angiotensin-(1–7) (ang-(1–7)), or directly ang-II to ang-(1–7) [47]. It is widely acknowledged that ang-(1–7) acts through G protein-coupled receptor Mas (MasR) that is expressed in many tissues including kidney, heart, brain, liver, and lung. However, there are some evidence that it can execute its action via other receptors including AT-2R [48] and MAS1-related G protein-coupled receptor D (MrgD) [49]. In general, alternative ACE-II/ang-(1–7)/MasR axis antagonize effects of ang-II in the cardiovascular system. Ang-(1–7) via MasR is responsible for decreasing the blood pressure, causing vasodilatation, improvement in renal blood flow and prevention of adverse tissue remodeling (Table 1) [50–52]. Ang-(1–9) is known since 2011 as endogenous ligand of AT-2R and its activity is connected with blood pressure decrease, protection from inflammation, fibrosis, and cardiac hypertrophy [53–55].

Last but not least, component of RAAS is an adrenal steroid hormone—aldosterone—produced mainly by adrenal glands and released in response to ang-II [56]. Aldosterone binds to the nuclear mineralocorticosteroid receptor (MR) in many tissues, mostly in the kidneys, and is responsible for control of secretion of potassium, sodium ions and water reabsorption, blood pressure rise and control of extracellular fluid volume. Moreover, aldosterone can promote heart remodeling and fibrosis [57].

Apart from the classical RAAS pathways, there are locally synthesized RAAS compound in many tissue and organs, e.g., in the heart, brain, kidney, or blood vessel. They can work separately or simultaneously with systemic RAAS peptides to maintain tissues homeostasis in case of growth and metabolism but also can contribute to some pathologies, including cancer [58, 59].

Broad physiological action of RAAS and its involvement in various cardiovascular diseases resulted in the fact that RAAS-targeting drugs are widely use in clinical practice. They have found application in treatment of arterial hypertension, heart failure, or nephropathy [60–62]. RAAS inhibitors include few groups of drugs: angiotensin-converting enzyme inhibitors (ACEIs), angiotensin receptor type 1 blockers (ARBs, also called sartans), aldosterone antagonists, and direct renin inhibitors (Fig. 1). ACE inhibitors suppress conversion of ang-I to ang-II. That inhibition leads to accumulation of ang-I and its alternative cleavage resulting in high production of ang-(1–7) [63]. Ang-II receptors blockers act via blockade of AT-1R and inhibition of signaling pathways associated with this receptor. Generally, they decrease activation of RAAS but contrary to ACEIs do not decrease levels of ang-II but specifically block only AT-1R. Normal levels of ang-II with concomitant blockage of AT-1R receptor can lead to increased binding of ang-II with AT-2R or enhanced conversion of ang-II into ang-(1–7) and activation of ACE2/ang-(1–7)/MasR axis. Altogether, ARBs exert its action not only by inhibition of AT-1R but also via activation of opposite pathways associated with stimulation of AT-2 and Mas receptors.

The broad action of RAAS components in cardiovascular system and multiple therapeutic strategies of RAAS suppression has led to extensive research on its role in various pathologies. Below, we summarize preclinical and clinical data on role of RAAS and its inhibitors in anthracycline-induced cardiotoxicity.

## Effect of anthracycline treatments on the renin-angiotensin-aldosterone system

### Effect of anthracyclines on RAAS gene expression

Doxorubicin can alter expression of various genes [64], possibly also the ones encoding elements of renin-angiotensin-aldosterone system, but the available data are very limited. Boucek et al. [65] have not found overexpression of the mRNA of RAAS genes in the hearts of rabbits treated with single dose of doxorubicin. Even though, levels of ACE mRNA were insignificantly 1.5-fold increased 4 days after doxorubicin injection but returned to control level after 4 weeks. Interestingly, the significantly increased ACE expression was noted in only one animal with myocardial injury, indicating that RAA pathway gene expression may be altered by doxorubicin, but the cumulative dose of the drug used in the study was too low to induce cardiotoxicity and observe genetic alterations. Results of this study may not be representative for long-term administration used in humans and should be validated in more relevant models with higher cumulative dose and multiple injections. Some changes, like increased dihydropyridine receptor (DHPR) or decreased atrial natriuretic peptide (ANP) mRNA expression can be persistent or even progressive after such low dose but probably for RAAS components longer stimulation would be necessary.

### Effect of anthracyclines on ACE/Ang-II/AT-1R axis

Based on the available data, it seems that increased activity of ang-II is one of the key events in AIC. Prominent ang-II action can be caused by increased ang-II synthesis or enhanced signaling from the ang-II receptors.

Firstly, doxorubicin causes increase of plasma ang-II level which can be even 3-times higher than in control animals [66]. Consistently, high levels of ang-II are also observed in the myocardium and paraventricular nucleus of hypothalamus (PVN)—one of the centers regulating cardiovascular system [66, 67]. Those findings implicate that in anthracycline-induced cardiotoxicity ang-II do not act solely in the myocardium and blood vessels but also modulates regulation of cardiovascular function at the central level.

There are multiple possible mechanisms leading to the increase of ang-II levels. There is some evidence that renin activity is increased in AIC causing higher degree of conversion of angiotensinogen to ang-I [68]. It has been reported that administration of single dose of doxorubicin caused significant induction in the levels of ang-I what can be considered as the surrogate for increased renin activity. Moreover, aliskiren, the renin inhibitor, ameliorated the rise of ang-I level [68].

Another mechanism is associated with increased ACE activity. Chronic DOX treatment led to significant, almost 2.3-fold increase of cardiac ACE activity in hamsters in comparison to that of the control animals [69]. Increased ACE activity can result from increased infiltrations of myocardium with macrophages that have ability to express ACE and which number is increased after DOX treatment [70]. Cardiac chymase activity is not affected by DOX; therefore, it is cardiac ACE, not the chymase, that plays the pivotal role in the RAAS activation and development of the AIC [69]. Baseline ACE activity could possibly predict the severity of the doxorubicin cardiotoxicity as it was observed for the renal-toxicity in rats, where higher ACE activity in the kidney was associated with stronger kidney damage [71]. Unfortunately, such correlation has not been studied for AIC. There is also no evidence on changes of ACE activity in the plasma or other organs so we cannot conclude whether the anthracyclines affect the systemic RAAS or only local network in the myocardium.

Ang-II elicits its action through its receptors AT-1R and AT-2R, whereas type 1 receptor is a crucial one and seems to play an important role in the AIC, even though data about changes in its gene expression elicited by doxorubicin are inconsistent. No differences in the AT1R mRNA expression were found in the rabbit model of AIC, however the dose of doxorubicin was relatively low [65]. Contrarily, doxorubicin increased the expression of AT1R mRNA and protein in a dose- and time-dependent manner in vitro in the embryonic cardiomyocyte cell line H9c2 [72] and in vivo in a rat model of cardiotoxicity [73]. Contrarily to AT-1R, type 2 receptor that mediates protective effects of ang-II is downregulated by DOX [73].

AT-1R overexpression is seemed to be promoted by the heat shock transcription factor 2 (HSF2) that, triggered by DOX, binds to the promoter region of AGTR1 gene at heat shock promoter element (HSE). During normal growth, HSF2 is localized in the cytoplasm as inactive form but even low dose of DOX leads to increased synthesis and translocation of HSF2 to the nucleus. Moreover, DOX, in the unknown manner, decreases HSF2 SUMOylation that normally prevents it from binding to HSE [74]. Additionally, DOX increases the MAPK-ERK signaling and lead to further activation of HSF2 and upregulation of AT1R [72]. Finally, DOX triggers translocation of the AT-1R to the cell membrane and binding with Gαq for downstream signaling transduction [72].

Role of AT-1R in AIC development was also proven in vivo in the genetically modified animals. AT-1R knock-out mice exposed to DOX have not developed any significant changes in LV internal dimensions and ejection fractions compared to mice with functional AT-1R that had enlarged left ventricle and reduced ejection fraction [75]. Structural changes in the cardiomyocytes, such as cytoplasmic vacuolization and myofibrillar loss nor apoptosis, have not been observed in mice with no functional AT-1R treated with DOX. Those observations were confirmed both, in the acute and chronic model of cardiotoxicity [75].

Increased amounts of ang-II and overexpression of type 1 receptor exerts different actions in the myocardium and can therefore lead to development of AIC. It is known that ang-II can induce apoptosis of ventricular myocytes via AT-1R independently of anthracyclines thus myocardial damage can result from direct anthracycline toxicity and ang-II stimulated apoptosis [72, 76]. Moreover, as mentioned before, ang-II via AT-1R can induce fibrosis and inflammation (Table 1) [38–41]. Cardiac fibroblasts responsible for matrix protein-production possess AT-1R on their surface, which after stimulation with ang-II activates mitogen-activated protein kinases (MAPKs) and extracellular signal-regulated kinases (ERK1/2) signaling that leads to overexpression of genes for collagen type 1 and 3, increase of fibroblast density and their proliferation [77, 78]. Also, ang-II-associated inflammation can trigger cardiac remodeling and fibrosis leading to dysregulation of hemodynamic function and finally to heart failure (Table 1) [79].

Additionally, high level of ang-II can induce cardiac cell stress, e.g., specifically oxidative stress, which can promote apoptosis and necrosis via the mitochondrial pathway. Increased production of free radicals is one of the best described mechanism through which DOX injures the myocardium. Some studies suggest that this effect can be modulated by RAAS [80]. Ang-II can induce production of free radicals, by activation of intracellular signaling processes [81] and enhance the direct effect of DOX that by dissipation of mitochondrial membrane potential and decreased ATP production leads to ROS generation (Table 1). It can be partially improved by ACE and/or renin inhibitors that have a strong effect on free radicals, especially by decreasing cytosolic total oxidant status and increasing total antioxidant status [80, 81] confirming involvement of ang-II in this process.

Besides altered RAAS function in the periphery, also some changes in its activity are observed in the central nervous system upon anthracycline treatment. Zheng et al. [66] observed that rats with AIC have higher levels of ang-II in the PVN what suggests that doxorubicin can modulate function of RAAS and cardiovascular system at the central level. It might be associated with increased number of COX-2 and CRH-positive neurons after doxorubicin. Oral inhibitor of COX-2 can decrease number of COX-2-positive neurons and normalize levels of myocardial, plasmatic and PVN Ang-II, attenuate myocardial injury, cell death and functional impairment of the heart caused by DOX, therefore suggesting that PVN COX-2 may be an intermediary step for PVN neuronal activation and excitatory neurotransmitter release, which further contributes to sympathoexcitation and RAAS activation in AIC. In the heart failure, upregulation of brain RAAS results in sympathoexcitation [82], increased thirst and salt appetite or activation of CRH- and vasopressin-containing neurons in PVN [83, 84]. Moreover, increased hypothalamic proinflammatory cytokines, e.g., produced by COX-2, activates RAAS and the HPA axis and contribute to sympathoexcitation [85]. Those preclinical data suggesting that anthracyclines induce sympathoexcitation at the central level correlates with clinical observations, where patients treated with anthracycline present overactivation of sympathetic system that can precede echocardiographic features of cardiotoxicity [86].

### Effect of anthracyclines on ACE2/ang-(1–7)/MasR axis

ACE2/ang-(1–7)/MASR axis possesses the protective function that can be modulated by anthracyclines. DOX causes significant decrease in ang-(1–7) levels and reduction in myocardial Mas receptor expression [73] which suggests that DOX can cause imbalance between both RAAS axes with overactivation of ACE/ang-II/AT-1R and decreased protective function of ACE2/ang-(1–7)/MASR axis**.**

In a model of doxorubicin-induced nephropathy expression of Mas receptor in kidneys was markedly reduced [87]. Downregulation of Mas receptor does not play significant role in the nephrotoxic mechanisms because mice with genetic deletion of MasR developed the same degree of toxicity as mice with normal receptor. However, treatment with losartan greatly increased by about 280-fold the expression of MasR mRNA in the kidneys of animals given doxorubicin and this renoprotective effect was not observed in mice with knockout MAS gene what can implicate that activation of signaling pathways of Mas receptor can provide renoprotection and possibly also cardioprotection [88]. In the AIC model, treatment with losartan increased ang-(1–7) levels without changes in the expression of MasR [73]. Local renin-angiotensin system can differ between various organs and can be differently regulate thereby the results from the studies on doxorubicin nephrotoxicity cannot be easily translated into cardiotoxicity mechanism and should be interpreted with caution.

### Effect of anthracyclines on aldosterone level and mineralocorticoid receptor

The data on the influence of DOX on aldosterone level and expression of its mineralocorticoid receptor (MR) are inconsistent. In one study, DOX induced a persistent increase of aldosterone levels already 2 days after the injection [89] in C57Bl6 mice while in another DOX did not affect plasma aldosterone levels nor cardiac MR expression in acute and chronic model of AIC [90]. The discrepancy in these observations may rely on mice gender differences, as female sex hormones can regulate aldosterone production and function.

Observed increase in plasma aldosterone level is probably caused by higher quantities of ang-II and increased stimulation of AT-1R [66]. Interestingly, high levels of aldosterone were associated with overexpression of AT-1R, suggesting that aldosterone promotes AT-1R expression in an MR-independent mechanism thus forming vicious circle of events aggravating cardiotoxicity [91].

To better evaluate the role of aldosterone and mineralocorticoid receptor in AIC, Lother et al. [90] used cardiomyocyte-specific inactivation of the MR gene in a mouse model. They have found that LVEF, LV contractility and relaxation were significantly impaired in the wildtype but not in the MR-deficient mice after doxorubicin treatment. Moreover, DOX-induced cardiac atrophy but not fibrosis was prevented by cardiac myocyte MR deletion what was also observed when MR was inhibited by eplerenone, an oral MR blocker [90]. The data suggest involvement of aldosterone and MR in AIC. Pharmacological MR inhibition had a beneficial effect on interstitial fibrosis what suggests that impairment of left ventricular function is mostly independent from the presence of cardiac fibrosis. Cardiac fibrosis seem to be pre-dominantly regulated by MR expressed on endothelial cells and macrophages rather than MR in cardiac myocytes thus this effect is observed only with pharmacological inhibition [90, 92].

## RAAS inhibitors in the prevention of anthracyclines-induced cardiotoxicity—preclinical studies

### ACEIs

The primary action of ACE inhibitors is inhibition of ACE activity and thus blockade or reduction in conversion of ang-I to ang-II resulting in decreased stimulation of AT1R receptors. Therefore, ACEIs lead to the reduction of total peripheral resistance, decrease in blood pressure, decreased afterload and thus increased stroke volume. Additionally, ACE inhibition through reduction of ang-II production decrease activation of intracellular signaling pathways from ang-II receptors.

Possible mechanism of ACEIs action in the prevention of AIC include the following: direct modulation of the cardiac and systemic renin–angiotensin system by inhibiting ang-II formation, reducing vascular resistances and then reducing myocardial afterload, indirect action against degradation of bradykinin and consequently enhancing NO synthesis, preserving sarcoplasmic reticulum Ca^2+^ homeostasis and thus the contractility of the myocardium [93–95]. In the animal models of AIC, pretreatment or co-treatment with ACEIs led to decrease of mortality, improvement of hemodynamic function, reduction of hypertrophy, decrease in level of serum markers of myocardial damage, or heart failure induced by doxorubicin [69, 96]. Similar observations have been noted for majority of available ACEIs, however some variations are observed. It is caused mostly by additional functions besides blockade of ACE that results from differences in the chemical structure. Based on their molecular structure ACEIs can be divided into three groups: dicarboxylate-containing agents (enalapril, ramipril, perindopril, lisinopril), sulfhydryl-containing agents (captopril, zofenopril) and phosphonate-containing agents (fosinopril).

*Enalapril* is probably the most extensively studied ACEI that showed efficacy in preventing AIC in various animal models [80, 97–99], but the data are inconsistent. Generally, enalapril do not protect against acute cardiotoxicity but have protective effect in chronic AIC induced by repeated small doses of Dox administered on a weekly basis [89]. Enalapril used in the dose of 10 mg/kg/day, for 1 week before, during, and 3 weeks after DOX, significantly attenuated the decrease in percent fractional shortening and prevented the doxorubicin-associated reduction in respiratory efficiency and cytosolic ATP content [98]. Importantly, enalapril abolished also the robust doxorubicin-induced increase in free radical formation. Interestingly, increase in cardiac apoptosis (measured by caspase-3 and -9 activity) was not significantly attenuated by enalapril [98]. This stay in line with another study, in which enalapril failed to normalize sub-acute daunorubicin-associated decrease in hemodynamic parameters and did not prevented absolute ventricular mass loss and weight loss [99]. However, QT prolongation, basal cardiac cell shortening and impaired catecholaminergic response were completely prevented by enalapril [80, 98]. Those data suggest that enalapril has only limited protective action on early development of anthracycline cardiomyopathy in rats.

The enalapril treatment comes along with increased phosphorylation of Akt and increased activation of PI3K, mTOR, and S6, when compared with Dox-treated mice what suggest that enalapril acts via activation of pro-survival AKT/PI3K pathway [89]. Enalapril exerts a protective effect not only by decreasing ang-II/AT-1R-mediated responses but also upon improving mitochondrial function in doxorubicin-treated rats and maintaining mitochondrial O2 consumption at control levels, preventing the depletion of cellular ATP content, and lowering the mitochondrial free radical leak [98]. Pretreatment with ACE inhibitor *captopril* or *enalapril* significantly reduces the thiobarbituric acid reactive substances concentration in the heart and ameliorate the inhibition of cardiac superoxide dismutase activity, suggesting that captopril and enalapril possess antioxidative potential that may protect the heart against doxorubicin-induced acute oxidative toxicity. This protective effect might be mediated, at least in part, by the limitation of culprit free radicals and the amelioration of oxidative stress [100]. Captopril contains in its structure a thiol and sulfhydryl group and thus can act as a free radical scavenger because sulfhydryl compounds are able to neutralize oxygen radicals by either a hydrogen donating or electron transferring mechanism [101].

*Perindopril* (2 mg/kg/day) used concomitantly with doxorubicin in Wistar rats did not improve DOX-induced heart dilatation, but enhanced antioxidant defense [102]. No improvement in cardiac function when treated with perindopril may be caused by too short treatment period or inappropriate animal model because rats in this study did not develop typical cardiotoxicity but rather mild cardiac dysfunction.

*Zofenopril* is an angiotensin-converting enzyme inhibitor characterized by a remarkable uptake by cardiac tissue, producing a striking and long-lasting inhibition of cardiac ACE as compared to other drugs of this class [103]. It is known to accumulate intracellularly thanks to its lipophilic structure. Moreover due to the presence of a sulfhydryl group it can act as ROS scavenger [104]. Sacco et al. [105] evaluated the level of ACE inhibition by *zofenopril* and *lisinopril* in the myocardium and in the plasma, depending on the dose. Both, zofenopril and lisinopril produced a dose-dependent inhibition of serum and cardiac ACE in rats. Zofenopril at lowest dose of 0.1 mg/kg/day showed a significantly greater inhibition of angiotensin converting enzyme in the myocardium than in the serum (Δ 20%), indicating a tropism for cardiac tissues and for myocardial ACE. Zofenopril, at a dose 0.1 mg/kg/day which partially inhibit ACE (app. 50%) and did not affect hemodynamics, almost totally prevented the QT lengthening induced by chronic administration of doxorubicin. For comparison, lisinopril was ineffective at this dose and higher dose of 10 mg/kg/day was required to achieve the same effect. Other groups confirmed the observation regarding zofenopril in the prevention of ST segment in ECG [106]. Moreover, zofenopril also prevented the depression of the inotropic response to isoprenaline in DOX-treated animals [106]. Zofenopril is more potent in the prevention of cardiotoxicity than enalapril or valsartan [107]. Presented data suggest that even low doses of zofenopril can be effective in the prevention of DOX-induced changes in ECG; however, authors did not evaluate the effect on the degree of apoptosis or ROS production. The lowest dose of zofenopril used in these experiments (0.1 mg/kg) is relatively close to that used for treatment of hypertension in humans.

Preventive effects of zofenopril or captopril lies partially besides the ACE inhibition and is based on the specific signaling pathways controlling cell survival through H_2_S, originating from sulfhydryl group. It was proven in various cardiovascular disease that H_2_S donors cause vasodilatation [108], exert anti-inflammatory responses [109], and protect from hypoxia/reperfusion damage in the heart [110].

While most research concentrate on the prevention of doxorubicin toxicity in the myocardium, Monti et al. [111] evaluated ACEIs in prevention of vascular damage. Zofenopril, in contrast to other ACEIs like captopril, lisinopril, or enalapril, can reverse the negative effect of doxorubicin on endothelial cells. When endothelial CVEC cells were exposed to various concentrations of doxorubicin in vivo, impaired cell survival, ERK1/2 related p53 activation and promotion of apoptosis by and induction of caspase-3 cleavage bypassing mitochondrial ROS production were observed, and could be prevented by zofenopril [111].

Co-treatment with *fosinopril* prevents from hemodynamic and morphologic changes in the heart induced by DOX [96]. Moreover, it partially reverses increase of cardiac enzymes levels (AST, LDH, CPK, cTnI) in plasma [112]. Importantly, fosinopril has an ability to attenuate DOX-induced decrease of sarcomplasmatic reticulum uptake of calcium ions and Ca2+-stimulated ATPase activity. Impairment of calcium homeostasis is most probably mediated by decreased expression of SERCA2 and phospholamban in sarcomplasmatic reticulum, what can be ameliorated by fosinopril [96]. Also Maeda et al. [112] reported that impaired calcium transients can be restored in isolated neonatal rat cardiomyocytes by concomitant treatment with ACEI. Ability to prevent remodeling of the cardiac SR membrane and attenuation of changes in myocardial Ca2+ homeostasis seems to be one of beneficial mechanism of ACEIs. This suggest involvement of ang-II in DOX-mediated downregulation of SERCA2 and phospholamban, but this mechanism has not been studied yet. Restoration of calcium ions homeostasis may significantly improve prevention and treatment of AIC because calcium ions regulate both systolic and diastolic function.

### ARBs

Angiotensin receptor blockers (ARBs) are the drugs that are antagonists of AT-1 receptors. They prevent binding of ang-II with AT-1R and inhibit intracellular signaling pathways from this receptor. However, the amount of ang-II produced by ACE remains at the same or even increased level; thus, it can bind with AT-2R or be conversed to other forms, e.g., ang-(1–7). The general effect of ARBs is similar to ACEIs, even though it can be more pronounced due to increased additional signaling from AT-2 and Mas receptors [113, 114].

Various ARBs have been tested in the preclinical models of anthracycline-induced cardiotoxicity with similar effects; however, some variations between specific drugs were observed, like those found in ACEIs.

Treatment with oral *candesartan* (5 mg/kg/day) started 4 weeks after the last dose of daunorubicin and continued for 4 weeks has resulted in the reduction of animals mortality from 50 to 19%, reduction of elevated blood pressure, LVP and LVEDP, increase of fractional shortening and E/A ratio, normalization of ventricular weight/body weight ratio, decrease of percentage of apoptotic cells in myocardium and amelioration of decreased SERCA2 mRNA expression when compared to control group [115].

*Telmisartan* (10 mg/kg/day) is also effective in prevention of AIC [73, 116, 117]. Iqbal et al. [116] evaluated the effect of telmisartan in the pre- and post-treatment model. In the pre-treatment model telmisartan (10 mg/kg/day) was administered orally 5 days before and 2 days after single injection of 20 mg DOX, while in the post-treatment model, it was administered only for 7 days after DOX. Pre- and post-treatment with telmisartan significantly attenuated AIC, however elevated tissue malondialdehyde MDA level and decreased level of glutathione GSH were normalized only by the pre-treatment with telmisartan. Histopathological examination revealed that signs of myocardial injury, such as high numbers of inflammatory cells, focal necrosis of muscle fiber, hemorrhage, and congestions, could be prevented by pretreatment with telmisartan while mild peripheral necrosis was noted in the post-treatment group [116]. This implicates that to obtain the best cardioprotective effects ARBs should be used before, or at least during DOX treatment, not only after or when the signs of AIC become evident.

The mechanisms of telmisartan-induced protection against Dox-induced toxicities may be partially AT-1R-independent, mostly via inhibition of lipid peroxidation and protection against GSH depletion, possibly owing to its lipophilic and antioxidant structure [118]. Besides blocking AT-1R, telmisartan poses additional partial agonistic activity on PPAR-γ which is known to have anti-inflammatory and antioxidant activities [119]. Protection before DOX-induced iNOS expression seems to be a significant factor in the telmisartan cardioprotection because iNOS overexpression leads to release of NO that promotes redox cycling and production of ROS [117, 120]. Moreover, Dox-induced apoptosis is associated with the increased expression of the endothelial nitric oxide synthase [121].

In another study, animals received *losartan* (30 mg/kg/daily) for 6 weeks concomitantly with doxorubicin [73]. Treatment with losartan attenuated deterioration of left ventricular function caused by DOX in similar level to telmisartan. Moreover, losartan significantly suppressed the upregulation of AT1R. Interestingly, telmisartan and losartan were not able to prevent decrease of ang-(1–7), MasR, and AT-2R [73]. Losartan decreased serum level of TNF-a, probably by inhibition of the ang-II ability to induce production of TNF-a by monocytes, macrophages, and vascular smooth muscle cells [122].

Losartan (0.7 mg/kg/day) was also tested in the combination with quercetin (3,3,4,5,7-pentahydroxy flavone), the flavonoid present in a variety of foods including vegetables, fruits, and wine [123]. The above combination resulted in more pronounce cardioprotection than losartan alone, most probably due to ability of quercetin to inhibit ACE via binding to its active site and reducing the conversion of ang-I [124].

Sakr et al. [125] evaluated different protocols of treatment with *valsartan* (10 mg/kg/daily): pre-treatment (2 weeks of valsartan followed by 2 weeks of doxorubicin), concomitant treatment, or post-treatment (2 weeks of doxorubicin followed by 2 weeks of valsartan). Concurrent or post- but not pre-treatment with valsartan of doxorubicin-treated rats reduced the cardiac enzymes serum levels, attenuated the oxidative stress, improved hemodynamic parameters, prevented from changes in ECG, ameliorated apoptosis and improved cell senescence. Importantly, there was no difference between concomitant and-post treatment use of valsartan, but the short duration of the study and treatment strongly limits the translation of this results into humans, where treatment with doxorubicin lasts for few months.

Treatment with *olmesartan* (10 mg/kg/day) for 12 days concomitantly with daunorubicin reversed worsening cardiac function, elevation of malondialdehyde (MDA) level in heart tissue, and decrease in the level of total glutathione peroxidase activity in the male SPRD rats [67]. Furthermore, ARB treatment downregulated matrix metalloproteinase-2 (MMP-2) expression, myocardial expression of ang-II, attenuated the increased protein expressions of p67 phox and Nox4, and reduced oxidative stress-induced DNA damage [67]. The reduction in the levels of MDA in the heart tissue of olmesartan-treated rats suggests that it protects myocardium against DOX induced lipid peroxidation.

Normalization of MMP2 expression by olmesartan is important observation because previous studies have shown that anthracyclines upregulate it by increased stimulation via AT-1R [126, 127]. Activation of MMPs can be one of mechanism leading to cardiac remodeling, dysfunction, and increase of cTnI due to its proteolysis, as observed in other cardiac diseases, like myocarditis or inflammatory cardiomyopathy [128, 129].

Also, *fimasartan* significantly improved survival of doxorubicin treated animals, protected from the ejection fraction decline and cardiac remodeling in dose dependent manner in the rat model. Effects were more pronounce when higher doses were used (10 mg/kg/day vs 5 mg/kg/day) [130].

### Aldosterone antagonists

Aldosterone antagonist, such as spironolactone or eplerenone, antagonize action of aldosterone at mineralocorticoid receptor. Spironolactone is the first and most used drug in this class; however, eplerenone is much more selective than spironolactone on target, but somewhat less potent. These drugs are widely used in the treatment of hypertension but they also exert protective action in treatment of heart failure. They exert positive effects on preventing cardiac fibrosis and remodeling induced by heart failure and myocardial infarction, which conclusively reduces the risk of both morbidity and death [131]. Considering that fact, there were few animal and human studies evaluating cardioprotective effects of aldosterone antagonists in AIC.

Spironolactone can prevent deterioration of systolic and diastolic function as well as attenuate cardiac fibrosis and myocyte apoptosis caused by DOX treatment [92]. Moreover, the expressions of TGF-β1 which plays important role in the induction of cardiac fibrosis, increased after DOX treatment, is significantly reduced by coadministration of spironolactone [92]. In one of preclinical studies, eplerenone (200 mg/kg/day), when started 5 days before doxorubicin, prevented the impairment of left ventricular ejection fraction and contractility. In the acute model, eplerenone was able to attenuate the interstitial fibrosis but in the chronic model this effect was not observed [90]. In another study, eplerenone did not protect from the acute or chronic cardiotoxicity in male mice. Moreover, the observations suggest that eplerenone synergistically amplifies Dox-induced molecular changes via sustained release of aldosterone and possible crosstalk with the ang-II signaling resulting in higher expression of AT-1R and connective tissue growth factor (CTGF) [89]. Summing up, the data concerning aldosterone and its antagonists in the prevention of AIC in animal models are highly limited and inconsistent.

### Renin inhibitor

As mentioned before, increased plasma renin activity plays a role in DOX-induced cardiotoxicity; therefore, its inhibition should have a protective effect. Aliskiren, an oral, nonpeptide direct renin inhibitor was tested in a rat model of acute and chronic cardiotoxicity. When administered as pretreatment (100 mg/kg/daily) before the single dose of DOX, it significantly prevented DOX-induced increase of levels of ang-I, LDH lipid peroxidation malondialdehyde (MDA), suppressed the myocardial apoptosis, reduced mortality and maintained the rats near to the normal status [68]. The observation has been also confirmed in the chronic AIC model [132, 133]. Furthermore, aliskiren significantly restored the DOX-induced alterations in antioxidant defense, reduced glutathione and superoxide dismutase (SOD) [132]. Systolic and diastolic impairment can be restored by aliskiren even with lower dose of 50 mg/kg [80]. The results of aliskiren treatment were comparable to telmisartan [68]; however, it has some advantages over existing RAAS blockers because it does not have ACE-escape-like activity, prevents the formation of both ang-I and ang-II, and produces effective blockade of RAAS without the compensatory increase in the plasma renin activity. There was no clinical trial evaluating the effect of renin inhibition in patients treated with DOX so observations from the in vivo studies cannot be confirmed.

### Targeting the ACE2/ang-(1–7)/MASR axis

Above mentioned observations on the role of ACE2/ang-(1–7)/MASR axis in AIC has led to the studies analyzing the cardioprotective utility of targeting ACE2. Based on the findings that autophagy-deficient mouse embryonic fibroblasts overexpress ACE2 [134], the hypothesis that ACE2 provides cardioprotection by reduction of myocardial autophagy was proposed and tested [135]. Lai et al. [136] reported that treatment of SPRD rats with human recombinant ACE2 after doxorubicin-induced cardiotoxicity has significantly reduced mortality from 32 to 4% and improved echocardiographic parameters compared to non-treated animals. Similar observations were reported by Ma et al. [137] who obtained myocardial ACE2 overexpression by intramyocardial injection of ACE2 adenoviral vectors. Animals overexpressing ACE2 had significantly lower 4-week mortality rates associated with doxorubicin treatment compared to Mock group and group with control vector: 18.75%, 71.88%, and 75%, respectively. At the molecular level, ACE2 overexpression resulted in decreased levels of oxidative stress markers, inflammation, and lower myocardial collagen depositions. Markers of autophagy and apoptosis, which were significantly increased in AIC rats, were attenuated by recombinant ACE2 or cardiomyocyte transfection with cDNA for ACE2 [136, 137]. The key explanation of this is the fact that ACE2 overexpression has changed the proportion of RAAS components. Ang-II and ACE expressions were decreased whereas levels of ang-(1–7) were higher than in the control group [137]. The proposed mechanisms behind the protective action of ACE2 is decreased stimulation of AT-1R due to higher conversion of Ang-I and Ang-II to Ang-(1–7) that further acting via Mas receptor leads to activation of PI3K-Akt/AMPK pathways and inhibition of the ERK pathway, which have known activity in inhibition of cardiac apoptosis [138–140]. That stays in line with Liu et al. who showed that ang-(1–7) infusions could significantly attenuate the left ventricular dysfunction and myocardial apoptosis by downregulating the pro-apoptotic protein caspase-3 and Bax and upregulating anti-apoptotic protein Bcl-xl expression in the rat AIC model [141].

The other possible mechanism includes suppression of DOX-triggered overexpression of TGF-β1 and thus reduction of heart fibrosis and hypertrophy [137], as well as inhibition inflammation [137, 142]. It seems that the protective effects of ACE2 can be also mediated by the miR-30e, which expression was significantly decreased in the myocardium of AIC rats and effectively prompted by ACE2 overexpression [136]. Silencing of the miR-30e inverted cardioprotective function of ACE2 both at the molecular level and in the echocardiography [136]. Physiologically, miR-30e is a negative regulator for Becclin-1 [143], a functional protein that interacts with Bcl-2 and is regarded as a mediator of autophagy [144] which stays in line with hypothesis on the involvement of ACE2 in protection against myocardial autophagy.

It is worth mentioning that effects obtained by ACE2 overexpression are at some extend like those observed upon ACEIs. ACE inhibition leads to the accumulation of ang-I and activation of collateral pathway, including upregulation of ACE2 that converts ang-I to ang-(1–7). ACE2 activity can be increased not only by ACEIs but also by overexpression of its gene (e.g., by using adenoviral vector). Both approaches are enough to reduce degree of cardiotoxic effect of doxorubicin, however in case of some parameters ACEIs are less effective than adenoviral vector. The difference may be due to the fact that ACEIs inhibits ang-II synthesis catalyzed by ACE but cannot inhibit process catalyzed by chymase and may not completely inhibit RAAS in the hearts of animals with AIC. On the other hand, ACE2 cleaves ang-II into protective ang-(1–7) and reduce level of ang-II, exhibiting stronger effect than ACEI [137]. For example, animals receiving ACEI cilazapril had lower mortality than controls but higher than ACE2-overexpressing rats (46.88 vs 71.88% vs 18.75%) [137]. Based on those observations it seems reasonable to search for new strategies aiming at increasing ACE2 activity as cardioprotection against AIC. For the moment, beside studies with ACEIs, there were no trials in humans with therapies affecting ACE2. Use of viral vectors to overexpress ACE2 is highly limited in humans due to lack of evidence, ethical issues, and safety considerations, but in the future, it can become a groundbreaking strategy.

## RAAS inhibitors in the prevention of anthracycline-induced cardiotoxicity—clinical studies

There is a variety of studies about AIC prevention that reported different outcomes with different drugs in diverse patients’ population. Thus, several meta-analyses are available. In 2015, Kalam et al. [145] has analyzed 14 original papers (12 randomized controlled trials and 2 observational studies) on different preventive strategies reporting that risk of cardiac events during or after chemotherapy was significantly reduced by RAAS antagonists (RR 0.11, 95%CI 0.04–0.29, *p* < 0.0001) as well as dexrazoxane, beta blockers, and statins. Those observations were confirmed later in Bayesian network meta-analysis conducted by Abdel-Oadir et al. [146] in which RAAS antagonists were the most efficient drug for the cardiotoxicity prevention with 84% risk reduction (OR 0.06, 95%CI 0.01–0.24) corresponding to a number-needed-to-treat of 9.9 [146]. In another meta-analysis of 6 randomized trials on prevention of early onset cardiotoxicity, BB and/or angiotensin antagonists were associated with significantly higher LVEF after chemotherapy completion (mean difference 6.06%, 95% CI 0.54–11.58, *p* = 0.03) [147]. Exploratory subgroup analysis proved that cardioprotective agents were beneficial in LVEF preservation in patients with higher anthracycline cumulative dose (doxorubicin > 300 mg/m^2^, epirubicin > 500 mg/m^2^) (mean difference 14.61%, 95% CI 12.26–16.97%, *p* < 0.001). Moreover, starting cardioprotection before or after chemotherapy show significant difference in LVEF (*p* = 0.002) and cardiac events (*p* < 0.001) favoring pretreatment [147]. Contrary, the analysis of 4 studies (324 patients) showed no significant difference in change of LVEF during chemotherapy (weighted mean difference 4.74, 95% CI − 12.6–3.1, *p* = 0.24) and in incidence of heart failure (OR 0.24, 95% CI 0.03–1.73, *p* = 0.16) between patients receiving ACEIs or placebo [148].

Some studies have analyzed combination of different protective strategies, mostly RAAS antagonists with β-blockers. The exploratory subgroup analyses comparing monotherapy to combination treatment of β-blockers and/or angiotensin antagonists revealed no significant subgroup differences, suggesting that prophylaxis with either a β-blocker or an angiotensin antagonist may be enough to prevent ventricular dysfunction [145, 147].

Generally, there is high heterogeneity between studies, most of them is performed on small number of patients, with varying methodology, use of nonrandomized design, different definitions of cardiotoxicity, lack of long-term measures of clinical efficacy, and low rate of events so the statistical power to detect small changes is insufficient. For the more complete image on the use of RAAS inhibitors in the prevention of AIC, below we summarize the finding of major studies on this topic, stratified by the class of drug.

### ACEIs

First evidence on the ACEIs protective role against AIC comes from the retrospective analyses of patients with various tumor types treated with doxorubicin. Comparison of cases that developed the cardiotoxicity with patients who did not have revealed that concomitant ACEIs use had a preventive role with a 73% reduction of risk of the LVEF decline [149]. Those observations were confirmed later by Wittayanukorn et al. [150] who using the Surveillance, Epidemiology and End-Results-Medicare-linked (SEER) database selected 6543 woman with breast cancer that received anthracyclines or trastuzumab. Patients exposed to ACEIs, defined as these who have filled prescription before or within 12 months after the initiation of anthracyclines/trastuzumab (*n* = 508) were compared with non-exposed group that has never had ACEIs prescribed (*n* = 6034). Patients receiving ACEIs had 23% lower risk of developing cardiotoxicity and 21% lower risk of all-cause mortality, even though those patients seemed to be less healthy with higher range of preexisting cardiovascular risk factors and more comorbidities. Starting ACEIs ≤ 6 months after the initiation of chemotherapy and exposition over 6 months were also associated with the decreased risk of cardiotoxicity and mortality [149].

More relevant data representing higher level of evidence comes from the prospective clinical trials. Georgakopoulos et al. [151] has investigated the incidence of clinical and subclinical AIC with preventive usage of enalapril (*n* = 43), metoprolol (*n* = 42), or placebo (*n* = 40) in 125 lymphoma patients treated with DOX, at mean cumulative dose ranging from 287.5 to 387.5 mg/m^2^. They have not found any significant differences; however, the study population was relatively small. The OVERCOME trial was conducted to verify efficacy of enalapril (max 10 mg twice daily) and carvedilol (max 25 mg daily) in the prevention of the anthracycline-induced left ventricular systolic dysfunction in the specific cohort of 90 adult patients diagnosed with hematological malignancies—patients with newly diagnosed acute leukemia who underwent intensive chemotherapy, or with relapsed/refractory Hodgkin’s and non-Hodgkin’s lymphoma or multiple myeloma undergoing chemotherapy before autologous hematopoietic stem-cells transplantation [152]. The cardioprotective drugs were started simultaneously at least 24 h before the first chemotherapy dose and were continued for 6 months after randomization [152]. At 6-month follow-up, changes in LVEF were observed only in the control group with significant LVEF decline of over 3%. Compared to controls, patients receiving enalapril and carvedilol had a lower incidence of the combined event of death or heart failure (6.7% vs. 22%) and of death, heart failure, or final LVEF < 45% (6.7% vs. 24.4%). Despite promising results that simultaneous enalapril and carvedilol cardioprotection prevents the LVEF decline, the study has serious limitation due to open-label scheme, no administration of placebo in the control group and between-group differences at randomization [152]. Specific population receiving intensive, high-dose chemotherapy regimens is also a limiting factor in generalization of the results.

Another randomized, single-blind, placebo-controlled study evaluated the cardioprotective effect of enalapril in 69 adult patients diagnosed with malignances requiring doxorubicin-based chemotherapy (60 breast cancers, 6 Hodgkin’s lymphomas, Wilms tumor, lung cancer and bone sarcoma) at cumulative dose ranging about 360 mg/m^2^ [153]. Enalapril (5–10 mg twice daily) was administered at least 24 h before the first cycle of anthracycline’ treatment and continued for 6 months. The study confirmed that enalapril seems to have important role in preventing DOX-induced systolic and diastolic heart dysfunction. Specifically, it prevents the LVEF decline, LVESV increase and alterations in E/e’ ratio, e’, and s’ velocity. Moreover, enalapril prevented the elevation of troponin I when analyzed 1 month after initiation of chemotherapy. Importantly, enalapril protects against decrease of s’ velocity [153], that was proved in previous studies to be a predictor of changes in LV systolic function after chemotherapy [154].

In a study performed by Cardinale et al. [155], 114 cancer patients who experienced troponin I elevation closely after high-dose anthracycline chemotherapy were randomized to receive enalapril 20 mg daily for 1 year (*n* = 56) or be placed in the control group (*n* = 58). Authors have found that the primary endpoint, defined as the absolute LVEF decrease > 10% with absolute decline below 50%, has occurred in 0% of patients receiving cardioprotection in comparison to 43% of controls. Moreover, enalapril protected against increase in the end-diastolic and end-systolic volumes. Risk of cardiac events was 30 times lower in patients treated with enalapril altogether proving the protective role of ACEIs in AIC. Later, the same team conducted the biggest study on enalapril in the prevention of AIC—the International CardioOncology Society-one trial (ICOS-ONE), a randomized, controlled, open-label, multicenter trial verifying two preventive strategies: enalapril (10 mg twice daily) administered in all patients starting before anthracycline-based chemotherapy (intervention arm, *n* = 136) and enalapril started only in patients with an increase in the troponin level during or after chemotherapy (troponin-triggered’ arm, *n* = 137) [156]. Enalapril was administered for 1-year period. Based on the trial results, in case of low cumulative doses of anthracyclines in adult population with low cardiovascular risk, there is no difference between two enalapril administration strategy regarding TnC elevation as cardiotoxicity marker. Authors of the study conclude that troponin-triggered strategy for the introduction of cardioprotective agents is more convenient [156]. However, in our opinion, considering the fact that troponin level can be elevated even after low dose of anthracyclines and do not correlate well with the incidence of cardiotoxicity, the administration of protective agents independently from troponin level would be more appropriate and more practical in clinical settings.

There was also one small, randomized, double-blind, placebo-controlled trial with enalapril (0,1 mg/kg/day once-daily from the first day of chemotherapy for a period of 6 months) in a group of 84 children patients (age 2–16) diagnosed with leukemia or lymphoma and treated with anthracyclines regiments at a cumulative dose ≥ 200 mg/m^2^ [157]. After 6 months, a reduction of mean LVEF was observed in both groups, but in patients receiving enalapril, the decline was significantly lower (3.5% vs 8.7%). Moreover, LVEF decrease ≥ 20% was observed in 3 patients in placebo group compared with none in studied group. Levels of cTnI and NT-proBNP were significantly lower in the enalapril group. Children are very vulnerable group and anthracycline cardiotoxicity in this group may be associated with significant complications thus studies evaluating protective strategies are of high importance. The study showed the positive effects of enalapril against AIC; however, the follow-up period was very short and longer observations are awaited.

Besides enalapril, there are also some observations with other ACEIs. Small prospective, single-arm study evaluated protective effects of ramipril or/and bisoprolol in 35 non-Hodgkin lymphoma patients with high risk of anthracycline cardiotoxicity [158]. The results were compared with results in the historical group of 62 high-risk NHL patients treated without cardioprotection. Lower frequency of new-onset clinical symptoms of cardiac damage (2.8 vs. 24.1%), no cardiac systolic dysfunction (0 vs. 8.5%), prolonged survival (projected 5-year overall survival 74 vs. 60%) and reduced 3-year mortality (0% vs 14.5%) were significant benefits coming from use of ramipril and bisoprolol. However, the results of the study must be interpreted with caution because the study group had more risk factors of anthracycline cardiotoxicity, less favorable International Prognostic Index and higher frequency of liposomal doxorubicin use than historical cohort what made groups not fully comparable [158]. Radulescu et al. [159] performed prospective study on 68 oncological patients treated with epirubicin regimens and perindopril and a gender- and age-matched group of 68 patients not receiving perindopril. At the end of chemotherapy, LVEF was found to be less altered in the patients receiving perindopril than in controls. However, perindopril could not prevent diastolic dysfunction and QTc lengthening.

Among currently ongoing trials the most awaited is the SAFE trial (NCT2236806). It is randomized phase 3, four-arm, single-blind, placebo-controlled study in 480 non-metastatic breast cancer patients treated with epirubicin- (cumulative dose from 225 to 600 mg/m^2^) or doxorubicin-based (cumulative dose 240 mg/m^2^) regimens with or without trastuzumab in the adjuvant settings [160]. Patients are divided into 4 groups receiving: bisoprolol (5 mg twice daily), ramipril (5 mg twice daily), both substances (each per 5 mg) or placebo. All patients will receive cardioprotection for 1 year or until the end of adjuvant treatment with trastuzumab, when it is recommended. All patients undergo cardiac surveillance with biomarkers (TnI and NT-proBNP), echocardiogram, and speckle tracking strain at baseline and every 3 months, until 2 years. Primary endpoint is a maximum change in LVEF. Protocol of the study seems to be promising but no results has been published yet.

### ARBs

Nakamae et al. [161] performed a pilot study on a small group of 40 patients with non-Hodgkin lymphoma treated by cyclophosphamide, doxorubicin, vincristine, and prednisolone (CHOP regimen) who were randomized to receive valsartan (80 mg/day) or placebo simultaneously with chemotherapy. The primary endpoint was the acute cardiotoxicity induced by anticancer treatment. After 1 week of observation, authors observed a significantly reduced increase of BNP (*p* = 0.001) in patients receiving valsartan, compared to control group. Apart from it, ARB inhibited the dilatation od LV and prolongation of QTc interval and QTc dispersion. Additionally, in this study, chemotherapy regimen did not affect the LVEF so the study did not allow an accurate assessment of the protective effect of valsartan on the systolic function of the heart [161]. The main weakness of this study is a very short observation time (7 days), which does not allow the appropriate evaluation of valsartan in the protection of AIC which rather occur as a late event.

In a study performed by Cadeddu, Dessi et al. [162–164] a group of 49 patient treated with epirubicin was randomized to receive telmisartan (40 mg/day) or placebo, for 1 week before the start of chemotherapy and continuing for the entire period of EPI treatment. The authors [162] did not note significant changes in LVEF and deceleration time in any of 2 arm throughout the whole treatment. One week after the second and third dose of chemotherapy, they observed a significant LV diastolic impairment, represented by a reduction in the early and late diastolic peak velocity (Em/Am) ratio at pulsed wave Doppler in the control group, which was effectively prevented by telmisartan. Decrease in the strain rate peak, an echocardiographic equivalent of early myocardial systolic dysfunction, which may be detected in epirubicin-treated patients long before a clinical manifestation of heart failure [165], was significantly prevented by telmisartan after third and fourth dose of cytostatic treatment [162]. While at the 12- and 18 -month of follow-up, the Em/Am ratio returned to the baseline in the placebo and telmisartan group, the differences between SR changes in both arm were still significant [164]. Effect of telmisartan was observed also on the serum level of ROS and proinflammatory IL-6, which were lower than in placebo group during the treatment suggesting additional anti-inflammatory and antioxidant properties of telmisartan [164]. Small and selected study population and relatively short follow-up period are the main limitations of this study.

The biggest clinical trial evaluating angiotensin antagonists in the prevention of AIC was PRADA trial [166] conducted on 120 breast cancer patients receiving adjuvant anthracycline-based chemotherapy. The study showed that concomitant treatment with candesartan prevented LVEF decline associated with adjuvant therapy—in the placebo group, the decline was 2.6 percentage point and 0.8 percentage point in the experimental group. Authors did not note any differences in right ventricular ejection fraction, as determined by MRI, neither in the left ventricular peak systolic global longitudinal strain by two-dimensional speckle tracking imaging. It is important to highlight that the reduction of LVEF was lower than authors were originally anticipated, so the power of these study was reduced to detect the differences between groups. The researchers have also measured and compared the levels of cardiac troponins I and T, BNP, NT-proBNP, C-reactive protein, and galectin-3 between placebo and candesartan groups. The median levels of all biomarkers increased from baseline until completion of anthracycline, but only levels of troponins and CRP were proportional to the dose of anthracycline. The ARB did not influence the level of these biomarkers [167]. The PRADA trial has analyzed also effects of metoprolol in monotherapy and in combination with candesartan; however, only candesartan was proven to be cardioprotective. The clinical utility of this observation is unclear because the increased level of troponin did not correlate with systolic or diastolic dysfunction. Generally, results of the PRADA trial seem promising but longer follow-up is necessary to determine whether candesartan will prevent the late cardiotoxicity.

### Aldosterone antagonists

There is only one study in humans, conducted by Akpek et al. [168], evaluating the protective effect of aldosterone antagonist, spironolactone at dose of 25 mg/day. Eighty-three female patients diagnosed with breast cancer and treated with anthracyclines were subjected to receive spironolactone or placebo, administered 1 week before the start of chemotherapy and finished 3 weeks after the end of cytostatic treatment. The study showed that a decrease in LVEF (app. 1.5% vs 14%) and lateral e’ velocity were significantly lower in the in the group treated with spironolactone than in the control arm. The increase of the level of the myocardial injury markers like troponin-I and CK-MB, was lower in the spironolactone group. The data suggest that spironolactone protects systolic and diastolic function, prevents myocardial injury, and has an antioxidative effect against anthracycline-induced oxidative stress; however, the study population was small and the observation period relatively short. This is the only study testing aldosterone antagonist in AIC. Another phase 2/3 clinical trial evaluating the effect of eplerenone in women receiving doxorubicin (NCT01708798) have been terminated due to futility.

### Guidelines and recommendations

Based on the results of conducted clinical trials, international cardiological and oncological societies have recently published guidelines on prevention, diagnosis, and treatment of chemotherapy-induced cardiotoxicity. Canadian Cardiovascular Society, American Society of Clinical Oncology nor European Society of Cardiology does not recommend to introduce cardioprotective strategy in all cancers patients treated with anthracyclines [4, 169, 170]. All of them recognize the potential protective effect of angiotensin antagonists, beta-blockers, and dexrazoxane but due to shortage of high-quality evidence, they do not recommend it in every patient. However, Davis et al. [169] suggested alternative prevention strategy based on 3 rules: (1) proper cardiovascular risk stratification before the chemotherapy and cardioprotection only in high-risk cardiotoxicity patients, (2) the early identification of cardiotoxicity, and (3) immediate introduction of proven therapies in patients with diagnosed cardiotoxicity. Similar recommendations have been made in the European Society for Medical Oncology (ESMO) consensus recommendations on management of cardiac disease in cancer patients throughout oncological treatment [171]. ESMO experts recommend that prophylactic use of ACEIs or ARBs and/or selected BBs may be considered in patients with a normal LVEF and cardiovascular risk factors who are scheduled to undergo anticancer therapy with known cardiotoxic agents (level of evidence II,B) [171].

In conclusions, most of the analysis proved the protective role of angiotensin antagonists but these findings need to be further validated in larger controlled randomized studies with more stress on the clinically relevant end points like death and development of clinical heart failure. Longer follow-up periods are necessary to precisely define the extent of cardiotoxicity prevention achieved with RAAS inhibitors. There is also need for better characterization of high-risk patients and conducting clinical trials in these cohorts.

## RAAS inhibitors in the treatment of anthracycline-induced cardiotoxicity

Treatment of anthracycline-induced heart failure is usually performed according to the same guidelines as non-chemotherapy associated HF thus diuretic, β-blockers, and RAAS blockers are administrated. There is no prospective clinical trials evaluating use of RAAS blockers in the AIC treatment; however, its beneficial effects have been reported in several retrospective analyses [172–176].

Lipshultz et al. al [174] analyzed retrospectively medical histories of 18 childhood cancer survivor’s to evaluate effects of enalapril use as a treatment of doxorubicin-induced cardiotoxicity. Enalapril was introduced due to symptomatic or asymptomatic heart failure with mean time from chemotherapy completion of 7 years. There was observed advancing improvement toward normal values of LV end-diastolic dimension, LV afterload, LV fraction shortening, LV mass, and systolic blood pressure in the first 6 years of enalapril administration. Unfortunately, all these parameters declined between 6 and 10 years of follow-up. By the 6 years of enalapril treatment, 6/6 symptomatic and 3/12 asymptomatic patients experienced cardiac death or underwent heart transplantation [174]. Those observations suggest that enalapril-based improvement in LV functions in long-term childhood cancer survivors treated with doxorubicin regimens are not permanent but early introduction of ACEI can slow down the deterioration of hemodynamic parameters. Moreover, when heart failure has developed some changes in the heart structure are irreversible thus complete cure may not be achieved. Probably enalapril does not address the primary defect in heart but rather reduces afterload by reducing blood pressure.

Cardinale et al. [173] focused their attention on the treatment of anthracycline-induced cardiomyopathy in a study was performed in a group of 201 mainly breast cancer survivors with a LVEF ≤ 45% primarily treated with anthracyclines regimens who subsequently received enalapril (mean daily dose 12 ± 6 mg) and when possible carvedilol concomitantly (mean daily dose 14 ± 7 mg). During the follow-up, 42.5% patients were classified as responders (LVED has normalized > 50%), 13.5% as partial responders (LVEF has increased of 10 absolute points but not raised above 50%), and 45% as non-responders (LVEF has increase less than 10 points). Responders have experienced less cardiovascular events than other groups. Multivariate analysis delivered that a short time-to-HF treatment and low NYHA stage were identified as the only independent predictors of LVEF recovery. Each doubling in time-to-HF treatment declined 4-fold the chances of complete cardiac recovery [173]. LVEF normalization and cardiovascular adverse event decline caused by anthracyclines may be obtained with early detection and promptly implicated HF therapy. Due to use of two drugs, it is the difficult to clearly identify which drug, enalapril or carvedilol, is responsible for the therapeutic effect. Also, other reasons, like second-line chemotherapy, can influence the HF treatment response.

Recently, Ohtani et al. [176] reported 67% rate of LV systolic dysfunction recovery among patients who developed anthracycline-induced cardiotoxicity. Majority of patients with AIC received RAAS inhibitors with or without β-blockers as the standard HF treatment. Multivariate analyses confirmed that early introduction of standard HF strategy is independent predictor of recovery (OR 9.39; 95% CI 2.27–52.9, *P* = 0.0014). Interestingly, some patients (10 of 33) recovered spontaneously without any HF medications but this cohort had generally milder LV dysfunction comparing to patients treated with anti-HF drugs. Also, Jensen et al. [175] observed the recovery of cardiac parameters after 3 months of ACE-I therapy (ramipril or enalapril) in patients with DOX-induced LVEF decline. Moreover, in their study the LVEF remained stable on ACEIs during median 33 months follow-up.

In a mini case series, Sheppard et al. [177] reported that sacubitril/valsartan (100 or 200 mg/day) can increase LVEF in patients who developed clinical heart failure or an asymptomatic LVEF decline inducted by anthracyclines. This intervention can also alleviate dyspnea. The authors noted the normalization of RV and LV systolic function and NT-proBNP level after 6 months of treatment. There were only 2 cases reported so the evidence is very weak.

Generally, it is recommended to use ACEIs or ARBs as the first-choice drugs of treatment of AIC. Cardiologists, who initiated Heart Success Program in the MD Anderson Cancer Center, recommends that promptly identification of cardiotoxicity and implementation of HF therapy (ACE-I, ARB) should be always attempted in all cases of anthracycline-induced cardiomyopathy [178]. Other cardiological societies recommend treating AIC according to general recommendations for heart failure [4, 169, 170].

## Summary and future perspectives

**Fig. 2:**
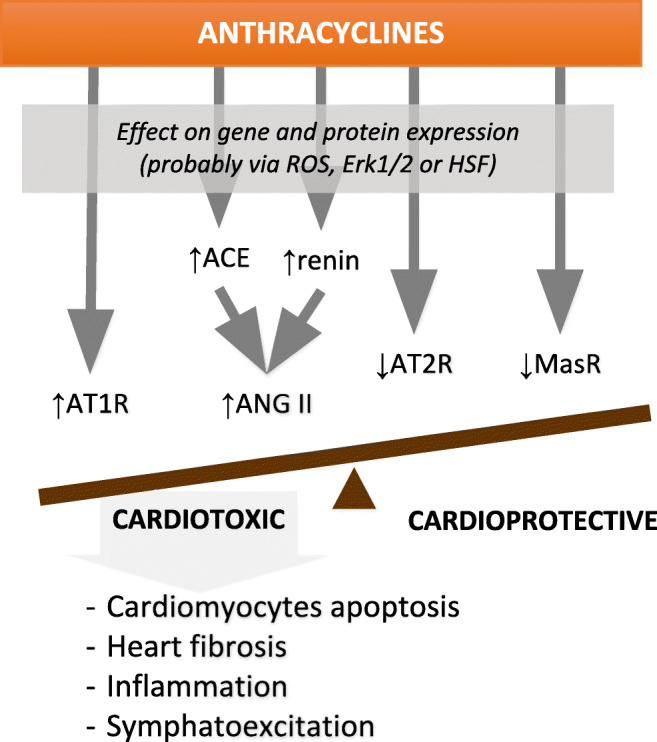
Possible mechanism of anthracyclines action on renin-angiotensin-aldosterone system. Anthracyclines leads to increased levels of angiotensin II (ANG II), overexpression of angiotensin type 1 receptor (AT1R), and decreased expression of MasR and AT2R receptors. This is caused probably by affecting gene expression via reactive oxygen species (ROS), Erk1/2 kinases or heat shock transcription factor 2 (HSF2). All those changes lead to imbalance of cardioprotective and cardiotoxic factors in favor of cardiotoxic

Based on the available preclinical and clinical data, it is undoubtful that renin-angiotensin-aldosterone system is affected by the treatment with anthracyclines and plays a role in the pathogenesis of cardiotoxicity. The evidence shows the activation of the ACE/ang-II/AT-1R axis, with significantly increased plasma renin activity, ang-II levels, and upregulation of AT-1R; nevertheless, the exact mechanism leading to those events is not yet clear (Fig. 2). It might be caused by modulation of RAAS gene expression by anthracyclines, but this hypothesis needs further confirmation.

The involvement of RAAS in the mechanisms of cardiotoxicity is also confirmed by studies with RAAS inhibitors that are effective in prevention and treatment. Moreover, interesting observations on this issue comes from the study by Guglin et al. [179] who found that lisinopril and carvedilol prevented trastuzumab (anti-HER2 antibody) cardiotoxicity only in patients previously treated with anthracyclines suggesting that RAAS system is disturbed by anthracyclines. Those results suggest that appropriate prevention of AIC may also protect from trastuzumab cardiotoxicity, but this should be further analyzed.

Most of the studies on RAAS in anthracycline-induced cardiotoxicity have concentrated on the evaluation of RAAS inhibitors as the preventive strategies. Results from various studies in animals and humans showed that ACEIs or ARBs are effective and can prevent or reduce intensity of cardiotoxicity. Different drugs have been tested with some minor differences between them. Based on the experiments in animals, it seems that, among ACEIs, zofenopril is the most effective in prevention of pathological changes in the myocardium as well as in the endothelial cells, probably due to its high affinity to cardiomyocytes and additional antioxidant activity. However, it has not been tested in any randomized clinical trial.

Different drug use settings (pre-treatment, concomitant, post-treatment) for AIC primary prevention with RAAS inhibitors has been evaluated, and the evidence supports its use concomitantly or even before anthracyclines. Administration of RAAS inhibitors after completion of anthracycline-based chemotherapy was associated with lower efficacy; however, those are the observations from the preclinical models of cardiotoxicity that need further validation in clinical trials. There is evidence from clinical studies by Cardinale et al. that primary prevention in all patients has same efficacy as prevention only in patients with early symptoms of heart damage, e.g., in the form of troponin increase. Both strategies are effective in various degrees, but we must be aware that not only efficacy but also toxicity, cost-effectiveness of screening for early symptoms, and cost-effectiveness of preventive strategies must be taken into account. Thus, in the view of currently available preclinical and clinical data, the exact recommendation on the most appropriate scheme of pharmacological prevention of AIC is not possible.

Enalapril, perindopril, ramipril, valsartan, telmisartan, candesartan, and spironolactone has been tested in clinical trials proving that they possess some protective effect, mainly preventing the hemodynamic abnormalities induced by anthracyclines and detected in echocardiography (Table 2). Not all studies have proved that RAAS inhibitors lead to reduction of risk of cardiotoxicity or heart failure; however, the follow-up in those studies was relatively short. Considering the late onset of AIC, longer observation is necessary and updates of trial results after longer-follow up are awaited. Other drawbacks of available clinical trials results are use of different endpoints and lack of consistent definition of the cardiotoxicity, what impede comparison between studies. More consistent trial design and use of unified definition could improve the level of evidence on AIC prevention.

**Table 2:** Summary of clinical trials evaluating angiotensin-converting enzyme inhibitors, angiotensin receptor type 1 blockers, and aldosterone antagonists in prevention of anthracycline-induced cardiotoxicity

Study	Chemotherapy type	Cancer type	Protection	No patients	Follow-up (months)	LVEF baseline	LVEF post chemotherapy	Cardiotoxicity n (%)	Heart failure n (%)	Death n (%)	Other findings
[151]	Doxorubicin	Hodgkin’s lymphoma Non-Hodgkin lymphoma	Enalapril Metoprolol Placebo	43 42 40	30	65.2 ±7.1 65.7 ± 5.0 67.6 ± 7.1	63.9 ± 7.5 63.3 ± 7.4 66.6 ± 6.7	E 10, L 6 (23.3, 14.3) ^a^ E 7, L 2 (16.7, 4.8) E 3, L 0 (7.5, 0)	2 (4.7) 1 (2.4) 3 (7.5)	0 0 0	
[159]	Epirubicin	Different malignant tumors	Perindopril Placebo	68 68	12 12	58.48±6.12 59.46±7.12	57.09±6.48 50.09±6.48	N/A	0 0	0 0	
[155]	Epirubicin Idarubicin Daunorubicin	Acute myeloid leukemia Breast cancer Ewing sarcoma Hodgkin’s lymphoma Myeloma Non-Hodgkin’s lymphoma	Enalapril Placebo	56 58	12 12	61.9 ± 2.9 62.8 ± 3.4	62.4 ± 3.5 48.3 ± 9.3	0 ^b^ 25 (43.1)	0 14 (24.1)	0 2 (3.4)	significantly less arrhythmias in patients receiving enalapril
[157]	Doxorubicin Daunorubicin	Leukemia Lymphoma	Enalapril Placebo	44 40	6 6	65.73 ± 5.41 64.85 ± 4.94	62.25 ± 5.49 56.15 ± 4.79	0 3 (7.5) ^c^	0 0	N/A	Children population
[158]	Doxorubicin	Non-Hodgkin lymphoma	Ramipril and/or bisoprolol Control (retrospective group)	35 62	18 36	N/A	N/A	1 (2.9) ^d^ 15 (24.2)	N/A	0 9 (14.5)	Prolonged survival (projected 5-year overall survival 74 vs. 60%; p < 0.05) for patients with primary cardioprotection
[152]	Idarubicin Daunorubicin	Acute leukemia Hodgkin lymphoma Non-Hodgkin lymphoma Multiple myeloma	Enalapril and carvedilol Placebo	45 45	6 6	61.67 ± 5.11 62.59 ± 5.38	-0.17 (-2.24 to 1.90)^e^ -3.28 (-5.49 to -1.07)^e^	4 (9.5) 7 (19.)^f^	N/A	3 (6.7) 8 (17.8)	
[153]	Doxorubicin	Breast cancer Hodgkin’s lymphoma Wilms tumor Lung cancer Bone sarcoma	Enalapril Control	34 35	6 6	59.39 ± 6.95 59.61 ± 5.70	59.93 ± 7.83 46.31 ± 7.04	N/A	0 0	0 0	
[161]	Doxorubicin	Non-Hodgkin lymphoma	Valsartan (80 mg/d) Placebo	20 20	7 days 7 days	N/A 64.8± 5.4	N/A 63.7± 6.7	N/A	N/A	N/A	
[162,164]	Epirubicin	Breast cancer Endometrium cancer Non-Hodgkin lymphoma Non-small cell lung cancer Ovarian cancer Salivary gland cancer	Telmisartan Placebo	25 24	18 18	66±7% 66±5%	66±6% 65±7%	N/A	N/A	N/A	
[166, 167, 180]	Epirubicin	Breast cancer	Candesartan-placebo Metoprolol-Candesartan Placebo-placebo Metoprolol-Placebo	60 60	10-61 weeks	62.1 (61.0, 63.3) 63.2 (62.0, 64.4)	61.4 (60.2, 62.6) 60.6 (59.4, 61.8)	N/A	N/A	N/A	
[168]	Doxorubicin Epirubicin	Breast cancer	Spironolactone (25 mg/d) Placebo	43 40	6 months	67.0 ± 6.1 67.7 ± 6.3	65.7 ± 7.4 53.6 ± 6.8	N/A	N/A	N/A	

*LVEF* left ventricle ejection fraction

^a^E, early cardiotoxicity from baseline to 12th month of follow-up; L, late cardiotoxicity after 12th month of follow-up; defined as LVEF < 50% and > 10% LVEF reduction

^b^LVEF < 50% and > 10% LVEF reduction

^c^LVEF decline ≥ 20%

^d^Assessed as occurrence of symptoms of cardiotoxicity

^e^Differences in change in LVEF between the intervention and control groups; there was no clearly indicated values of LVEF after chemotherapy

^f^Heart failure or ≥ 10% decrease in LVEF

Despite some controversies about use of RAAS inhibitors in the primary prevention of anthracycline-induced cardiotoxicity, their wide use in clinical settings is highly possible in near future. Recent results of clinical trials are more in favor of primary prevention what is slowly reflected in the guidelines of leading oncological societies. At the end of 2019 European Society of Medical Oncology has published recommend that prophylactic use of ACEIs or ARBs and/or selected BBs may be considered in patients with a normal LVEF and cardiovascular risk factors who are scheduled to undergo anticancer therapy with known cardiotoxic agents [171]. There are also several ongoing clinical trials evaluating this topic (NCT02236806, NCT02818517), and their results may help to finally answer the question about efficacy of primary prevention and its clinical utility. In our opinion, in near future, RAAS inhibitors can become a standard prevention during chemotherapy with anthracyclines.

We must be aware that primary prevention of AIC with RAAS inhibitors can only reduce the risk to some extend and cannot eliminate it completely. Patients with previous cardiovascular diseases, even treated with RAAS inhibitors, will still have increased risk of AIC. Such patients require close monitoring during treatment and probably use of addition safety measures and treatment. Moreover, further research to understand mechanism of this type of adverse events and better preventive methods are awaited.

RAAS inhibitors are widely used in the treatment of various diseases and no serious adverse events are reported. Similarly, in the prevention of AIC, no significant side effects were reported. RAAS inhibitors do not interfere with the antitumor activity of doxorubicin [106, 111] and do not affect cancer cell proliferation or metastatic potential. In some cancer types, they are even considered as having positive effect on patients’ survival and treatment outcomes [181]; thus, their use in cancer patients is safe.

Concluding, there is growing number of evidences showing that dysregulation of renin-angiotensin-aldosterone system consist and important stage in the chain of events leading to cardiotoxicity but further studies in validated animal models and in patients are necessary to find its exact place and role. Clinical trials showed that protection with RAAS inhibitors is effective in some extent, but the quality of the data and level of evidence is still too low to include obligatory prevention for every patient treated with anthracyclines. Nevertheless, ongoing and planned trials can change the perspective. Moreover, novel strategies, like targeting ACE2/ang-(1–7)/MasR pathway, showed promising results in the animal models and in future can find their way to the clinic.

## Data Availability

Nor applicable.
